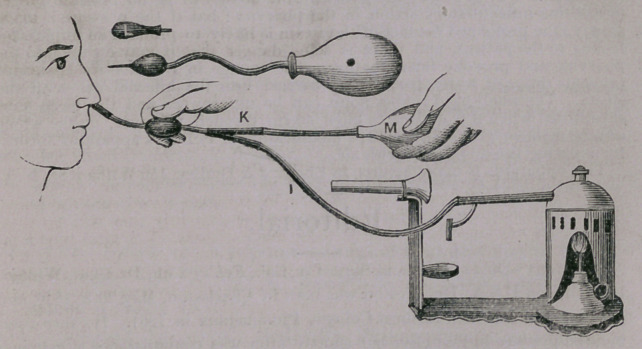# A New Method of Applying Steam to the Middle Ear and of Inhaling Medicated Vapor with a New Steam Atomizer

**Published:** 1872-05

**Authors:** 


### A new method of applying Steam to the middle ear and of inhaling medicated vapor with a new STEAM ATOMIZER.

"Inhalation has been employed from the earliest ages. It was used in Greece, in Rome, in Arabia, and from thence, with the extension of medical knowledge, it spreads every where, until finally the recent discovery of a method of subdividing liquids into a cloud or spray, and thus utilizing them to the purpose of Inhalation, has at once enlarged its sphere of usefulness, and given a fresh impetus to the study of the Therapeutics of Inhalation.

This Apparatus (Fig 255) consists of a nickel plated boiler with safety valve

A resting on a body B; the lamp C which heats the boiler; the metal arm E which holds the glass tube F; the arm D of the boiler into which the arm E is slipped; the medicine glass H; and the face shield G. This face shield of glass is flattened its *whole extent* which allows it to be introduced farther into the mouth, and presents gagging; and other unpleasant symptoms, which the round face shields are liable to produce. This atomizer is the most durable of any now offered to the profession. The boilor is nickel plated, and the body and shield holder are of polished brass, and consequently they will not rust or be affected by heat as readily as those made of iron. The glass tube F is arranged to slip into the arm E and is easy of adjustment or removal. If the straight glass tube should be broken, it can be replaced at one-sixth the cost of the right angled tubes used on other Atomizer's; or a tube may be formed by drawing out a piece of glass tube in the flame of a spirit lamp or gas flame, and then dividing it by a slight scratch with the blade of a knife. In this way you can obtain a supply of tubes without being compelled to send to the manufacturers for tubes of a peculiar shape.

Fig 256 shows the atomizer with the glass tube removed, and a flexible rubber tube attached to the point E connecting the boiler with the hard rubber face shield I. This shield has a chamber for the purpose of holding sponge, which can be medicated with any drug desired. If necessary reduce the flame of the lamp so as to maintain a low pressure of steam in the boiler. The steam passing from the boiler through the flexible tube into the face shield, and through the sponge, produces a warm steam or medicated vapor. It is so simple that a child can administer it without any trouble and is the surest and most convenient way that has been employed for administering warm steam or medicated vapor, as the face shield covers up the nose and mouth, and has an opening in the top for the exhalation and inhalation of the air, (the opening can be closed with a cork if desired). By this means the patient is compelled to inhale the steam or vapor.

Formerly when it was necessary to administer steam to children, the means employed was either, to stick a hot poker into a pail of water, and hold the child over it to inhale the vapor; or to hold the child up to the spout of the croup kettle to let it inhale, if it would, the escaping steam, very little of which entered the childs throat.

Sometimes the child would close its mouth, and inhale through the nostrils, in which case the steam could not enter the throat. but would condense before it reached it. The importance then of having an efficient apparatus is self evident. This apparatus will be most valuable in the treatment of Croup, Diphtheria, Asthma, Whooping Cough, etc.

Fig 257 represents the face shield removed, and Dr. D. B. St. J. Roosa's apparatus for blowing steam into the middle ear, substituted.

There are certain diseased conditions of the middle ear, in which the lining mucous membrance is thickened and somewhat sunken, the normal secretion of mucous is lessened or apparently absent: even the Eustachian tubes sharing in the geneaal thickening, and dryness perfectly pervious and permitting the free movement of air through them, known as Chronic Aural Catarrh of the variety is called Sclerosis. Tinnitus aurium is an irregular symptom of the disease, but often present and often distressing.

Catheterization and inflation after Politzers method sometimes improve the hearing slightly, more often not at all; neither does it improve the Tinnitus. Such cases are discouraging. It seems reasonable to infer that in such cases some application to the comparatively dry surface of the tympanic cavity which shall increase the amount of secretion, would prove a step in the right direction, toward reestablishing those conditions which are demanded by the laws of sound. Such an application is moist heat. It seems reasonable to expect from the use of steam some improvement in that distressing and obstinate symptoms, Tinnitus aurium, (which is caused by the irritation of the auditory nerve) because of the admitted sedative effect of moist heat.

In treatment of the diseases of the tympanic cavity, its condition of moisture or dryness should be considered on account of its relation to the acoustic requirements of the hearing apparatus, and when dryness exists, our therapeutic efforts should tend to reestablish the normal secretion, while on the contrary, astringent remedial agents are in place only when there is hyper secretion. The application of steam will reestablish the secretions in the abnormally dry tympanic cavity, and place the stretched membranes under more favorable acoustic conditions, and thus improve the hearing, while. through its sedative effect the irritated auditory nerve becomes quiet. Steam is applied in the following manner. The face shield I is removed and the Y shaped glass tube attached to the extremity of the flexible tube I. The soft subber bulb M is attached by a flexible tube to the opposite arm of the Y. To the remaining arm is attached Dr. Roosa's hard rubber apparatus. The steam passes from the boiler through the flexible tube I and the apparatus, which contains a piece of sponge. The extremity of this apparatus is inserted into the end of a hard rubber Eustachian catheter, already in the patients mouth.

Compress the rubber bulb M. This forces the steam through the Roosa apparatus and the Eustachian catheter into the middle ear. The pressure upon the bulb must be made quickly, and the point must be removed from the end of the catheter after each compression. If these directions are observed, no inconvenience will be felt by the patient, and there will be no possible danger of exciting inflammatory action in the pharynx; but if the pressure is made slowly, the prolonged contact of the steam is likely to produce an unpleasant feeling to the patient, and there is some danger that it may escape into the pharynx and provoke inflammation. The sponge in Dr. Roosa's apparatus prevents drops of water from being carried into the internal ear with the steam. The small hard rubber point can be unscrewed, and the nasal tube substituted when it is desired to blow steam through the nose, or the whole can be detached, and Dr. Roosa's modification of Politzers apparatus formed for inflating the middle ear with air.

This apparatus is manufactured by Shepard & Dudley, 150 William St., N. Y.

## Figures and Tables

**Figure f1:**
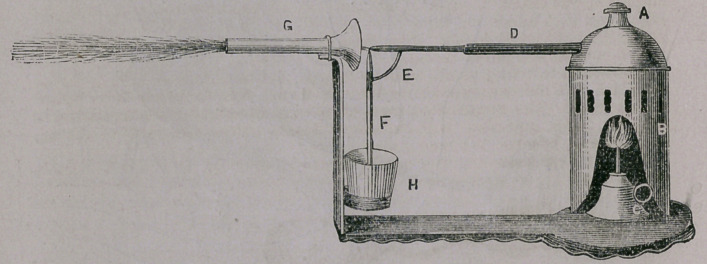


**Figure f2:**
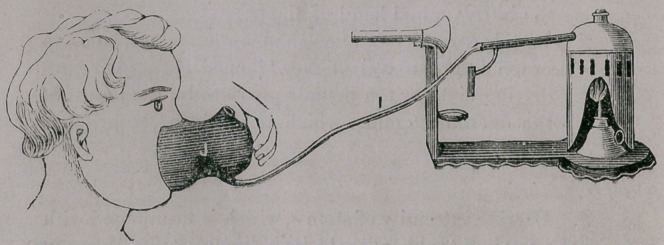


**Figure f3:**